# Pilot Evaluation of a Remote Psychotherapy Service for Students Who Self-Harm: University–Community Outpatient Psychotherapy Engagement (U-COPE)

**DOI:** 10.3390/ijerph21010103

**Published:** 2024-01-17

**Authors:** Joanne Worsley, Danielle Young, Paula Harrison, Rhiannon Corcoran

**Affiliations:** 1 Department of Primary Care and Mental Health, University of Liverpool, Eleanor Rathbone Building, Liverpool L69 7ZA, UK; corcoran@liverpool.ac.uk; 2 Department of Psychology, University of Liverpool, Liverpool L69 7ZA, UK; hldyoun2@liverpool.ac.uk; 3 Student Administration and Support, University of Liverpool, Liverpool L69 7XZ, UK; paula.harrison@liverpool.ac.uk

**Keywords:** self-harm, U-COPE, psychotherapy, remote provision, accessibility, therapeutic relationships

## Abstract

Self-harm is becoming increasingly common in student populations. Brief psychological therapies might be helpful for those who have recently self-harmed. The current paper reports on an evaluation of a brief psychotherapy service delivered via remote means, namely University–Community Outpatient Psychotherapy Engagement (U-COPE). The service combines elements of psychodynamic interpersonal and cognitive analytic therapy to help students who present with self-harm related difficulties. The primary aim was to understand students’ and practitioners’ experiences of a remote psychotherapy service. Semi-structured interviews were conducted with a total of nine participants (seven students and two practitioners). Interview data were analysed using thematic analysis. Analyses of the interviews across the stakeholders revealed three overarching themes: ‘Accessibility’; ‘Therapeutic experiences’; and ‘Spaces and places of therapy’. Students appreciated the rapid access to intervention, especially as student services are typically characterised by long waiting lists. Despite the brief nature of the intervention, many students reported feeling a sense of control over the direction and pace of the therapeutic sessions, which is an important consideration for those who self-harm. The findings suggest that U-COPE may be helpful to students with difficulties related to self-harm. Further investigation of this brief intervention is warranted in order to ascertain whether U-COPE has a long-term impact on difficulties and distress-related behaviours.

## 1. Introduction

Emerging adulthood represents a unique developmental period in young people’s lives [1]. Coinciding with this critical developmental period, the transition to university often involves young people relocating, assuming responsibility for their own academic workloads, developing independence, and managing their own finances [2,3]. Such pressures often leave young people particularly vulnerable to mental health difficulties [4]. Self-harm, defined as any intentional act of self-injury or self-poisoning regardless of motivation or suicidal intent [5], is increasingly prevalent among students [6]. As self-harm is indicative of psychological distress and difficulty [7], as well as associated with an increased risk of death by suicide [8,9], it is important for educational institutions to offer accessible and effective interventions for students with self-harm related difficulties [10]. 

There is a growing body of evidence demonstrating that psychological therapies can lead to a reduction in self-harming behaviours, as well as improvements in associated difficulties, such as depressive symptoms and suicidal ideation [11,12,13]. Systematic reviews and meta-analyses suggest that various approaches, such as Cognitive Behavioural Therapy, Dialectical Behaviour Therapy, and Emotion-Regulation Group Therapy, may be helpful for people who self-harm [11,12,13]. Brief Psychodynamic Interpersonal Therapy (PIT) has also been shown to be effective in reducing self-harm and suicidal ideation [14]. Although brief therapies are important in increasing access, the evidence base surrounding such therapies remains limited.

One example of a brief therapy is the Hospital Outpatient Psychotherapy Engagement (HOPE) service, which was set up in a hospital emergency department (ED) in response to high levels of self-harm in a disadvantaged area. The HOPE service offered brief psychological therapy, combing PIT and Cognitive Analytic Therapy (CAT), to people presenting to a hospital ED following self-harm. Combining PIT with elements of CAT may be helpful for people who self-harm as these approaches have a relational focus, considering a person’s relationships with others and the way they relate to themselves (e.g., self-critical, supportive, dismissive) as important processes underlying self-harm. While there is currently not strong evidence to support a specific brief psychotherapy approach, combining PIT with elements of CAT showed promise in an ED context [15]. Taylor and colleagues found that 64% of referred individuals attended at least one therapy session, and nearly half of those referred attended all four therapy sessions [15]. There was, however, evidence of possible difficulties with the alliance [15]. It is possible that a positive working alliance might have been harder to forge in this context, especially as individuals were aware of the brief nature of the therapy and therefore they may have felt less inclined to establish a therapeutic alliance given the limited number of sessions. This is an important consideration, especially as links have been made between the creation of strong alliances and successful therapeutic outcomes [16,17]. 

The HOPE service has recently been adapted for implementation within a university context, namely University–Community Outpatient Psychotherapy Engagement (U-COPE) service. In the face of the global COVID-19 pandemic, U-COPE was delivered exclusively via online means and consisted of an assessment, four weekly sessions of psychological therapy, and a follow-up session which was offered three weeks after the end of therapy. As the evidence base surrounding brief therapies remains limited, especially in relation to psychological interventions for self-harm which specifically target university students [4,18], this paper reports on an evaluation of a brief psychotherapy service for students who self-harm. Through qualitative means, the present evaluation aimed to understand students’ and practitioners’ experiences of a remote psychotherapy service. Specifically, we sought to understand the acceptability of the intervention and identify barriers and facilitators to effective intervention and implementation of the U-COPE service. A qualitative approach was selected as appropriate to provide a level of rich lived experience detail from the perspectives of those accessing or delivering the service that was sufficient to inform to nature of future delivery.

## 2. Methods

### 2.1. The U-COPE Service

Following referral, students are contacted by a U-COPE practitioner via telephone within 72 h and offered an initial appointment within two weeks of referral. The initial appointment involves a current assessment of mental health and a risk assessment. Following the initial appointment, students are offered four therapeutic sessions during which U-COPE practitioners combine PIT (a relationship approach focusing on relationships with self and others) with elements of CAT (such as the use of visual mapping and a focus on identifying solutions to difficulties). Using this integrative approach, practitioners work collaboratively with students to identify patterns or conflicts in emotional experiences and interpersonal relationships in order to formulate a shared understanding of these experiences and identify ‘exits’ or solutions to students’ difficulties. Following the final therapeutic session, there is a three-week gap which allows students to explore the skills they acquired during therapy. One final session is offered to students following the three-week gap. 

### 2.2. Participants

Seven female students and two practitioners (one male, one female) were purposively sampled. To participate in the evaluation, students had to be enrolled at one of two universities in Northwest England and have experience of accessing the U-COPE service remotely due to recent (within the past year) self-harming behaviours. With regard to recruitment, U-COPE practitioners circulated the participant information sheet to students during the course of therapy. When a student expressed an interest in taking part, the therapy innovation lead shared the student’s contact details with the research team. Practitioners were sent a copy of the participant information sheet and invited to participate via email.

### 2.3. Data Collection

Semi-structured telephone or video call interviews using prepared topic guides were conducted via online means in March 2021. Interviews, conducted by the first author, with practitioners explored their experiences of delivering the brief psychological therapy via remote means. Practitioners were given the opportunity to reflect on the perceived benefits of the intervention, as well as any challenges and/or difficulties. Example questions included: ‘what has your experience of delivering the therapy within the U-COPE service been like?’, ‘what do you think about the way U-COPE is structured and delivered?’, and ‘how do you feel the remote sessions would compare to face-to-face sessions?’. Interviews, conducted by the second author, with students explored experiences of accessing the U-COPE service via remote means, acceptability of the intervention, perceived benefits, and factors affecting the impact of intervention. Example questions included: ‘How did the service feel?’, ‘Were there enough sessions, too little, or too many?’, and ‘Did remote sessions impact your ability to get the most out of the service?’. Interview duration ranged from 25 min to one hour. All interviews were audio recorded and transcribed verbatim.

### 2.4. Data Analysis

The qualitative data were subjected to thematic analysis. Thematic analysis is a qualitative method that aims to identify, analyse, and report recurrent themes in data [19]. The six phases included familiarization, generating initial codes, searching for themes, reviewing and defining the themes, and naming the themes [19]. Line-by-line coding, undertaken independently by the first and second authors, ensured that data were not overlooked. The second author identified preliminary themes. In consultation with the final author, the first author refined and renamed the themes and subthemes. This process ensured that the final analysis did not reflect the personal interpretation of one team member. 

## 3. Results

Analyses of the interviews across the stakeholders revealed three overarching themes (summarised in Table 1).

**Theme 1: Accessibility**

***‘Quick and ‘easy’ referrals and access***

Many students were referred to U-COPE by University Mental Health Services. Those who experienced this method of entry reported that the referral process was ‘easy’ to navigate, which is an important consideration for those experiencing distress. Thus, many students reported positive experiences as they felt the responsibility was taken off them:

*The University Mental Health Department referred me. It was easy in the sense that I didn’t really have to do anything. I just had to agree to one email, and I was sent straight there.*

(SU1)

*I thought it was very accessible because they referred me and then all I had to do was fill in a form saying ‘I consent to this’ and then I was on.*

(SU2)

*It was quite easy, because I just phoned up the University Mental Health Services, and they referred me to them [U-COPE] straight away. I thought it was quite accessible, they gave me all the info[rmation] I needed so it was really good.*

(SU6)

Participants also reported that the referral process was exceptionally ‘quick’, especially in comparison to other student services, which are typically characterised by long waiting lists:

*I think it was really fast, probably the fastest I’ve ever seen, especially for students.*

(SU4)

*There’s often like a long waiting time. So, how quick it was, that was really helpful to me.*

(SU7)

*Ours is very quick in terms of how long they have to wait so within three days we contact them, and they are seen within a week or two. That’s very quick compared to most therapies where you are waiting for months.*

(Practitioner 2)

In fact, some participants stated that this exceeded their expectations of a service for students:

*I expected there to be a really long waiting list. It was probably about a month, maybe a bit less than that, but it felt really quick. I didn’t feel I was twiddling my thumbs waiting for help. It was a really positive experience.*

(SU6)

*It helped me because I felt it was well organised and quick to get me into seeing someone straight away… It was really well organised like the time it took at the start when I got my first appointment was so quick. I was surprised at that.*

(SU5)

*I was referred on the third and my first meeting was the fourth of February. It was so quick. I wasn’t expecting it at all, so it was really helpful for me.*

(SU2)

Practitioners reported that the speed at which students were referred and able to access the service was particularly reassuring for certain individuals, especially those who have ‘*a deep sense of not being cared for*’. The importance of rapid access to intervention in ensuring wellbeing and preventing escalation of distress-related behaviours was also highlighted, particularly for those who were actively self-harming. Immediate psychological support may reduce the risk of deterioration and short-term repetition:

*I think being contacted quickly, especially because they generally would have approached someone because they’re in crisis/in distress… to have that acknowledged fairly quickly, I think can be quite reassuring—you can prevent someone getting cold feet [and] it prevents someone from escalating if they are self-harming at the moment. [It] can be quite containing as well.*

(Practitioner 2)

***Remote access ‘made it more accessible’***

As students were not required to travel, many perceived online provision to be ‘more accessible’. As engaging online mitigates practical and psychological barriers to full engagement with services, remote provision may be beneficial for certain individuals, such as those with mobility issues that limit their ability to travel:

*Not having to travel—I do have a bit of mobility issues… It was readily accessible; this is probably the biggest positive.*

(SU1)

*I definitely was able to make [the sessions] more because it was online and the freedom of being able to do it wherever was really good. I wouldn’t have went as much if it was in-person so wouldn’t have got as much out of it.*

(SU3)

*I had more willingness to go every week because I didn’t have to go anywhere. Because it was just in my room, I didn’t have to go somewhere else. It made it a lot easier in that way not having to commit to going somewhere out of the house every week, especially if I was struggling.*

(SU6)

In fact, one student acknowledged that she had previously disengaged from a different mental health service due to the need to travel:

*When I’ve had sessions before, like with the HOPE intervention… I couldn’t make my session because I couldn’t get myself to the centre one day and I just stopped going because it was very hard to actually get there early when I wasn’t feeling great… I think it made it more accessible [referring to U-COPE being delivered remotely] and I managed to go to every session because in the past I’ve not managed to do that just because of issues surrounding not being able to get out the house.*

(SU4)

In line with this, the option to access the service remotely might be an important factor underscoring increased attendance and engagement:

*I don’t think we’ve got a very high rate [of students dropping out] … Maybe working at home has helped with that not having to go to the university building to be there on time for your session and just having to log online. Maybe that’s something that helps with attendance.*

(Practitioner 2)

**Theme 2: Therapeutic experiences**

***Therapeutic alliance***

Therapeutic relationships are one of the most important aspects of mental health care. In fact, therapeutic relationships constitute the essence of successful therapy as some students explicitly stated that their therapist, and the therapeutic relationship that they established within UCOPE, was ‘the main best thing about the therapy’:

*My therapist was fantastic. They were the main best thing about the therapy. They were easy to talk to. They did make it successful.*

(SU1)

*I think pretty much straight away we got on really well and I really felt like she understood me… I just think it was great, especially with my therapist being really, really understanding and empathetic. She made it successful for me.*

(SU6)

Many students highlighted positive attributes of their therapist (e.g., ‘*My therapist was really approachable*’ (SU7)), which enabled them to feel ‘comfortable’ (e.g., ‘*They were really like not informal but like chatty in a nice way that made me feel comfortable*’ (SU5)). However, some students acknowledged difficulties forming therapeutic relationships. When a positive therapeutic alliance was not established, the outcomes of therapy were harder to achieve, and this was illustrated by one student who struggled to open up during her sessions as she ‘*didn’t vibe with [her] therapist*’:

*I think the fact that I didn’t vibe with my therapist, that had an impact. I think I found it difficult sometimes when they asked me a question, I would just go silent because I didn’t really know what to say, because I felt like not that comfortable.*

(SU4)

As the therapeutic relationship is an essential component of successful therapy, U-COPE therapists reported initially believing that face-to-face sessions would be necessary to establish engagement and rapport and that they would find it hard to create the intensity of relationship required for trust and change without meeting in-person. Despite these early reservations, therapists did report that positive working relationships were formed in the context of remote working:

*It Is much better to have this kind of therapy In-person as it’s better for the relationship/better for the alliance, but generally that hasn’t been too much of a problem, not as much as I had anticipated. So, I think it’s generally been very, very positive.*

(Practitioner 2)

Students also envisaged difficulties forming therapeutic relationships online but were instead surprised to find that working remotely did not impact as much as they had initially anticipated:

*I was expecting it to be rather difficult [due to] it being online. I didn’t think much of a connection would have been, well as much of the connection would have been made.*

(SU1)

*I don’t think [online delivery] did impact it [referring to the therapeutic alliance]. They [referring to therapist] made being online as easy as they could have.*

(SU3)

On the other hand, however, some students encountered difficulties establishing a therapeutic alliance in the context of remote working. Remote provision impacted the therapeutic relationship for some students as they felt as though there was space to hide, whilst others reported that their therapist did not ‘pick up on things’ that they wanted to discuss:

*I think it made it a bit more difficult [referring to developing a rapport]. I’ve been to a few therapists, and… in-person is a bit more emotional for me. I can feel myself being a bit more emotional in-person… It feels weird to cry to a computer… [So] there were some limitations in place that did hinder forming of relationships… Communicating in an official capacity about mental health to someone not in the same room as me while I’m in my home [was challenging].*

(SU1)

*Seeing just this other person on screen I thought that was quite intimidating and you didn’t feel quite like a real connection… In terms of connecting with my therapist, I thought it was a bit more difficult because in a way [you] don’t see them as a real person, even though you know they are but it’s just like a video… I just think the dynamic I had with my therapist wasn’t very productive. They didn’t really pick up on things that I wanted to say as much as other people have in the past. I think maybe they weren’t the most suitable person for me, and then also it being online made it more difficult, because it’s easier to just sit there and not reply than it would be if I was actually with them… I think [in-person] would have made it easier to develop a bond with the person.*

(SU4)

In line with this, practitioners found it harder to ‘pick up’ or identify non-verbal forms of communication during remote sessions. As non-verbal communication is often relied upon to maintain engagement, practitioners emphasised that they feel ‘more connected’ when working with clients in a therapeutic space:

*You’re more connected in-person. You can read body language a bit better. It’s just something about the space. Where I used to work, the sessions were always in-person, and it felt a bit more connected. I just think it’s easier to pick up on things because I might say to someone, ‘you seem to recoil there, or your head went down’. So, I’ll notice body language but that’s a little bit harder to do online. And then you’ve got the lag issues, and then it’s trying to read the tone of someone’s voice. Things like that can be a bit trickier online. I don’t think it’s been a major impediment, but I do think you feel the connection stronger in-person.*

(Practitioner 2)

*Sometimes when you really want to feel the feelings in the room, sometimes that’s difficult to do… Sometimes it’s really hard to pick up on the subtle feelings in the room. It’s also a bit of a nuisance when there are connection issues because that makes that even harder… From other points in my career, I’ve learned the power of feelings in the room.*

(Practitioner 1)

Although alliances sufficient to facilitate psychological change appeared possible online, participants felt that this particular form of psychotherapy would ‘work better’ in-person. In light of such concerns, students and practitioners would welcome the option to engage in, or deliver, the therapy in-person:

*I would have preferred it to be in-person just because I feel like I can connect more with who I’m speaking to in-person.*

(SU5)

*I’d prefer it to be in-person. It just feels more personal, and I find it easier. I think the sense of collaboration is even easier to develop in-person.*

(Practitioner 2)

***Collaboration: “It’s not quite the same as working in-person’***

Collaboration forms a central part of this intervention. During the course of therapy, practitioners work alongside their clients to co-produce a ‘Staying Well Plan’ (known as a collaborative map illustrating triggers that lead students to experience self-harming urges and alternate ways of soothing or managing those feelings). Although many participants acknowledged that this form of psychotherapy would feel more collaborative in-person, this collaborative element was not lost in the context of remote working:

*Even though it was online, my therapist still managed to make it feel as collaborative as possible, which was very helpful.*

(SU2)

*When I went into it, I had low expectations, because where I’m from the mental health team is really, really bad… I felt like it would be more of her telling me how to do things, but when I actually got there, I found it was very much just talking to somebody who was really, really understanding about your feelings and stuff like that. So, it was not at all what I expected. I thought it would be more structured as in like, you tell the problem, they say how to sort it, but it was more like you worked through it together, which I really liked… We would have the map and rather than me just editing it, we would go through it together, so it was still collaborative. She would share her screen, and I would say things for her to edit in real time, so it was still very much a thing we did together, it just was online rather than on paper. Because sometimes, I wouldn’t have any idea how to put it into words so she was really good in that sense because I would explain things with metaphors and stuff, and she would word it correctly.*

(SU6)

Students reported that creating the collaborative map contributed to the person-centred and tailored nature of the therapy. The creation of this plan was found to be ‘very helpful’ in supporting students’ wellbeing during and beyond the therapy sessions as many felt a sense of ownership over it:

*It [referring to the ‘staying well plan’] was tailored… It’s one of the physical things that I’ve been given from therapy that was really made for me… It has given one very helpful tool to have, the thought map, which I have been using after discharge. It’s been very helpful.*

(SU1)

*I do keep coming back to it [referring to the map] because it’s just like a paper form of my brain and how I’m thinking, and it helps me to work through my thoughts and stuff. It’s been so useful to have it and it’s so personal as well.*

(SU3)

Nevertheless, the creation of the collaborative map using online software was found to be harder in the context of remote working as practitioners were not ‘comfortable’ or familiar with the software package. When working therapeutically in a shared space, practitioners and clients are in close proximity and would work in a side-by-side way, collaboratively constructing the map using pen and paper:

*You literally have a physical piece of paper in between you and the client you’re working with, whereas you might be sitting opposite them for a lot of the session generally, but for the map they will come and sit next to you so closer. So, you’ll look at the map together… If I’ve done an initial map, I might think, ‘oh, I’ve missed something off here’, which in-person that’s not a problem, you just scribble it out and go again. It’s harder to do online, even if you’ve got a programme where you can shift lines about in diagrams, I’m just not that comfortable using it. I’m not very quick with it. It doesn’t feel as though there is that space. It’s not quite the same as working in-person. But having said that, again, it’s not a major impediment. I just think it’d be slightly better [in-person]. It would feel more collaborative.*

(Practitioner 2)

Whilst working remotely, U-COPE practitioners explored different ways of constructing the map. One practitioner constructed the map before the session, whereas another constructed the map during the therapy session:

*Doing the map [online] has been difficult, because we’re meant to be there together in-person, and we’ll draw the map together, that’s been very difficult to do over the internet. I’m not quite sure of the best way to do that. So, I’ve been trying different things. I’ve been doing a preliminary map and bringing it to the session and working through it with them and that’s not ideal, but it has worked pretty well. But that’s been a limitation.*

(Practitioner 2)

*I use [Microsoft] OneNote, and I’ll share my page and more often than not, we will map it together. I mean they’ll be the odd one that finds it distracting or uncomfortable but in the main, we’ll have a shared page where we are constructing this map together.*

(Practitioner 1)

Although some students found this process collaborative, others felt it was quite ‘one-sided’ (e.g., ‘The creation of the plans to help being majority one-sided did probably limit what could be done’ (SU1) and ‘I wouldn’t say it was collaborative like at all’ (SU4)). In light of this, many participants reported that this aspect of the therapy would work better in-person:

*I would always prefer to have the sessions in-person myself, especially when it comes to work on the map. I think it’s a system that works better in-person.*

(Practitioner 2)

*He made that map, and then he was just asking me, ‘do you think there’s any more we could add?’ We properly went through it and he always asked me every step of the way. It was definitely collaborative… [but] it would have been a lot better obviously in-person.*

(SU3)

*The mapping as well would have been better in-person.*

(SU5)

***Efficacy***

Most of the students interviewed reported finding U-COPE to be effective. Students cited increased self-knowledge and the acquisition of new ‘tools’ in relation to promoting recovery and enhancing feelings of wellbeing:

*It [referring to the therapy] was able to accomplish its goal and it has given me new tools to help better myself.*

(SU1)

*I’ve learned so much about myself… This is the first time I’ve finished a course of therapy and feel like it was a complete thing. I feel like I’m ready to go it alone.*

(SU2)

*Being able to identify your triggers and why you think the way you do so it was a good and new experience… I’ve definitely learned a lot about myself from doing that.*

(SU7)

Students reported that U-COPE felt tailored and individualised (e.g., ‘U-COPE was helpful and tailored’ (SU1) and ‘It felt really good actually like it felt really about me [as an] individual… It felt very tailored to me’ (SU5)). In particular, many reported feeling a sense of control over the direction and pace of the therapy, which is an important consideration for those who self-harm:

*I think it was really well structured and I liked how it was based on my progress, rather than a set plan of where we have to be every week. I set the time of how I wanted to take things and set the pace, which was really good because it meant that it was less forced. It felt really individualised to me almost. It was about me and my progress.*

(SU6)

Some students made comparisons between U-COPE and other forms of therapy that they had previously engaged in:

*Other [therapies] I’ve had have been, ‘well you think this so don’t think this, you think this so don’t do this’, whereas this is like ‘this is where the feeling comes from. This is what you feel when this happens’. But in other therapies I felt like it was right session one, you write a list, session two, talk about what makes you anxious, session three, talk about this, whereas this felt very personal and tailored to what I needed which was great.*

(SU2)

*It was perfect really. After the first session, I was so happy. I couldn’t believe that finally, there was an actual therapy that was not just the same old listing off the symptoms of Web MD. It felt like it was actually helping me and having an impact… I feel like my thoughts are so much more valid. I feel so much more secure in myself and how my brain works. I’ve definitely learnt and gained so much from it.*

(SU3)

In comparison to other forms of therapy, U-COPE was regarded as unique as it offered students a space to talk through, as well as process, past experiences:

*[Students] seem to find it extremely beneficial and useful. I think it’s the psychodynamic aspect, which isn’t that common in mainstream therapy. To look at the past and make those links… Because we have such a big focus on that, that seems to be quite revolutionary for a lot of people who struggle with issues of self-harm and those issues are very often rooted in childhood experiences.*

(Practitioner 2)

*I think people seem to be struck by that space to explore the historical links, and to revisit the feelings of the past. I think there’s not many opportunities where they get to do that, and I think people view it as almost like a gift. It validates their difficulties in the here and now when they have that space to explore what happened in the past, and to explore how it’s affected them… The students that we see, generally speaking, have a very self-critical inner dialogue, a lot of self-criticism, a lot of shame, a lot of guilt, a lot of self-hatred. So, allowing them to see how this has started, it allows them to be more compassionate towards themselves.*

(Practitioner 1)

Although some students found the length of the therapy to be suitable to adequately address their needs (e.g., ‘I think that was the right amount. I did find myself starting to run out of things to say’ (SU1) and ‘I feel like I covered enough for it to be effective’ (SU5)), the number of sessions was not appropriate for all students, especially those seeking to delve deeper into childhood experiences. Thus, some students would have appreciated the option to extend the course of therapy (e.g., ‘I wouldn’t have minded maybe a couple more sessions’ (SU7)):

*A longer time scale would have been a lot better because the way he [the therapist] made you think about things, brought up other things like over time so maybe, if it was longer, then you’d be able to properly go into everything or maybe if you weren’t finished the therapy in the end, there could be an extra three sessions or something like that, just because I feel like there was a lot brought up but not enough time to talk about it all… There were times where I was like right ok, so we’ve only got six sessions. I could talk for years about how I feel and what’s going on in my head, but how do you stop it at six?.*

(SU3)

Particular concerns were raised in relation to those who take longer to ‘open up’ about difficult life experiences. Some students struggled to engage during the early stages of therapy and would have benefitted from additional sessions:

*I think it took me like a couple [of sessions] to feel safe to open up to another level… It wasn’t until almost the last session that I really managed to open up because I was quite nervous… I think if I’d have engaged better in the beginning, I’d have felt more comfortable with my therapist and opened up quicker then we could have gotten a bit further. I think because it took quite a few sessions, it wasted the limited time we had together.*

(SU4)

*A few people have been concerned about the length because they feel a bit more able to handle the self-harm, they feel like they know what’s going on, they feel like they’re on the right track in the right direction. But then sometimes they might feel there’s so many issues here, there’s so much from my childhood… Sometimes they get a bit frustrated I think that we’re coming to the end… Some people, not very often, will struggle to get there. There was one in particular I was thinking of, as we just didn’t really get there in four or five sessions. It was just the emotions couldn’t come into the session, very heavily guarded, history of childhood trauma… I think maybe some might need longer.*

(Practitioner 2)

Other students suggested that it would be beneficial for there to be a couple of additional sessions focusing specifically on changing behaviours:

*Having two or three more sessions to better understand like how to change behaviours instead of just talking about it and being aware almost… Having a few more sessions, some roundoff sessions where you can properly understand what you need to do next, more focused on exits rather than just awareness.*

(SU3)

*More information on how to stop doing behaviours and thoughts, instead of just being aware of them.*

(SU4)

**Theme 3: Spaces and places of therapy**

***Spaces of comfort and safety***

Engaging in therapy from their home environment enhanced feelings of comfort and safety for some students, which may have enabled them to feel more able and willing to discuss difficult experiences and/or articulate complex emotions:

*What it talks about is quite deep and quite personal and because I was in my bedroom in my safe space, I find it easier to talk about things rather than if I was going to his office or a different room… I felt comfortable in my space, and my room was how I like it. I felt like I could smell my home. So, I definitely preferred the remote sessions.*

(SU2)

*When I’ve had sessions before, like, with the HOPE intervention going in, I couldn’t make my session because I couldn’t get myself to the centre one day and I just stopped going because it was very hard to actually get there early when I wasn’t feeling great so that having it in my room, like a safe environment, was good.*

(SU4)

*I don’t really like to go to face-to-face appointments to talk about my emotions. So, for me, it was good to have it in the comfort of your own home.*

(SU7)

As well as familiarity with the environment, practitioners highlighted that young people often prefer communicating via online means. In fact, some students reported finding it easier to talk to a screen about difficult experiences and emotions rather talking to someone in-person. Communicating via remote means may enhance feelings of control and empowerment, which are important considerations for those who self-harm:

*I think being in-person it’s a little bit harder to talk about your feelings than it was talking to a screen so in that way I think it was easier.*

(SU6)

*One girl said to me recently that she might not have engaged if it had been face-to-face because she gets too self-conscious, but she feels more in control in her own environment.*

(Practitioner 1)

*Some prefer the online communication. Some say I’m much more comfortable, it feels much less clinical to be working online. Somebody said the other day that they’ve had therapy in the past, and they didn’t like going to the place where it was, they didn’t like sitting there with somebody as it just felt like going to a doctor. For them, being at home was quite empowering. They felt like they could open up more being at home.*

(Practitioner 2)

Nevertheless, not all students felt comfortable engaging in therapy from their home environment (e.g., ‘The other thing about being online is that this place, my room, this is a comfort place, and it’s weird to expose those things in this vicinity’ (SU1) and ‘I think [in-person] would have made it easier to develop a bond with the person and just being able to open up somewhere out of your own space’ (SU4)). Engaging in therapeutic sessions requires a private space where a client feels able to speak freely. However, some students highlighted privacy concerns, which impacted on their willingness to ‘open up’. This is particularly important in the context of shared student housing:

*[As] I live in a shared house at uni[versity], I felt not that comfortable being potentially in hearing space of other people, especially if it has to do with them so maybe that was one of the reasons I couldn’t open up as much and maybe being in an actual room with someone would have been easier for me to just like let everything out.*

(SU4)

Thus, some students would have preferred face-to-face provision, while others preferred engaging remotely; a ‘one size fits all’ approach may not be appropriate in the aftermath of the pandemic. A blended model of provision, with students being able to select the mode of delivery which best suits their needs, was suggested as a useful approach moving forward, especially as choice may enhance feelings of control:

*When the country starts to open up again properly, and everything obviously goes back to face-to-face, I’d probably recommend doing online and in-person, and a combination just so it gives people a chance to go to U-COPE if they want but also do it from the comfort of their own home, which I think a lot of people might be interested in doing this service if it was set up like that.*

(SU7)

*Once we’re working in-person, that’s something to bear in mind that some people prefer that approach. It’s maybe something we can keep–keep some online sessions for certain students who are much more comfortable working that way.*

(Practitioner 2)

***Moving the emotions on***

Although many students appreciated the opportunity to engage remotely, issues arose for others in respect of talking through difficult experiences and emotions in their own spaces. In particular, students raised concerns in relation to the conclusion of remote sessions, with some citing that there was ‘an atmosphere’ as difficult emotions were left ‘hanging around’ upon completion of a therapy session:

*I think exploring painful feelings can be difficult for people… I think sometimes people find it difficult after the session, because it all feels quite raw and intense.*

(Practitioner 1)

*There was a couple of weeks where it was really intense, and it was talking about a couple of traumatic things to me where I then would be emotional for the rest of the day. So, it would impact me negatively.*

(SU7)

It is possible that a change of scene might change the internal space, especially as concerns were raised as there was no physical separation or transition between a therapy session and ordinary life. The need to travel home following a therapy session is an important transition, providing a space to process emotions. Such journeys are transitions into a different way of being; however, this transitional space was lost when engaging remotely:

*It was difficult because there’s no physical transition to help you along with just changing your mindset. Just sat in the same chair, and you’re sitting with the same emotions that you’ve just dug up.*

(SU1)

*When I first did it, it did feel really weird. So, I’d end the call and suddenly it was silent, and I was on my own and all of the thoughts that we brought [up], that we talked about hit me. So, at first, it threw me off a little bit because it was some deep stuff, which was just a lot and talking to someone like that, you don’t really think about how it affects you until after when it’s silent. I got used to that so towards the end of my sessions, I didn’t feel like there was that much of an atmosphere, like I was fine, I could go back to what I was doing but at the start, that was something I struggled with.*

(SU6)

In light of this, one student highlighted the need for practical advice following the sessions:

*I think more practical advice and skills, especially after the sessions because often at the end [of a session], I was left just crying and didn’t really know how to manage my emotions. So, I think more practical advice to support and regulate emotions afterwards.*

(SU4)

## 4. Discussion

This paper reports on a pilot evaluation of a brief psychotherapy service for students who self-harm. Our qualitative findings fall into three main categories: accessibility, therapeutic experiences, and spaces of therapy. 

In the face of COVID-19, U-COPE was delivered remotely. Remote provision mitigated practical and psychological barriers to full engagement with the service. Students appreciated the rapid access to intervention, especially as student services are typically characterised by long waiting lists [4]. This is an important consideration, especially as immediate psychological support may reduce the risk of deterioration and short-term repetition. 

Despite the brief nature of the therapy, therapeutic alliances sufficient to facilitate psychological change appeared possible online. In line with previous research (e.g., [20]), when students forged a positive working relationship with their practitioner, this resulted in the therapy being regarded as successful. Many students found U-COPE to be a unique service as it provided a space to consider and reflect on interpersonal difficulties and/or traumas, often rooted in childhood experiences. Most of the students interviewed also reported feeling a sense of control over the direction and pace of the therapeutic sessions, which is an important consideration for those who self-harm. The collaborative process of co-creating the ‘Staying Well Map’ was also highlighted as an important element of the therapy as many students reported feeling a sense of ownership over this process and found this resource to be useful in aiding their recovery. Thus, these findings are in line with previous research suggesting that therapies with a relational focus, including PIT and CAT, may be helpful for people who self-harm (e.g., [14,15,21]). 

Consistent with previous research (e.g., [22,23]), some students suggested that remote provision increased comfort levels, whereas others felt their place of comfort and safety was tarnished. Some students were concerned that others would be able to overhear, which is particularly important in the context of shared student housing. As there is no physical separation or transition between a therapy session and ordinary life, students were left with strong feelings to manage alone in their home environment. Although these feelings subsided over time, previous research suggests that engaging in a transitional period following a therapy session improves mental wellbeing (e.g., [13]). 

Our findings should be considered in light of several limitations. First, as all interviews took place through virtual platforms, our sample may represent those who are more likely to be engaging well with virtual therapy. Second, a large proportion of eligible students did not participate in the present evaluation. In particular, as only a small number of female students participated, our findings only reflect the experiences of this particular group. The findings are therefore not generalizable to all students who engaged with U-COPE, especially male students. It should be noted that the views of male students may differ. Thus, the inclusion of a broader sample of students would have resulted in a greater understanding of the experiences of the intervention. Future research should therefore endeavour to incorporate the voices of male students using targeted recruitment strategies through trusted organisations and university societies. A larger-scale evaluation exploring the long-term effects of this brief intervention is also needed. Future research should include follow-up interviews to explore whether the effects of the intervention are sustainable over time. 

Notwithstanding these limitations, there are a number of recommendations. First, as many students reported a sense of being left with difficult emotions in their own environment following the conclusion of a remote session, guidance should be embedded around the importance of leaving the space in which the therapy session took place (e.g., going to make a cup of tea or spending time in a different room, having a plan of what to do straight after the sessions that initiates helpful transition). Second, as choice and agency are important considerations for students who self-harm, the option to engage with U-COPE via online means should be retained, especially as some students reported that they may not have engaged with the service if this option had not been available. Thus, remote provision is an important tool to reduce stress. Third, as many students highlighted the length of the therapy as problematic, greater flexibility in terms of approach would be beneficial, especially as the option to extend the course of therapy by a few extra sessions would have been welcomed by students. Taken together, presenting students with options, such as therapy length and engagement method, affords a sense of control. Finally, students emphasised the importance of co-creating the ‘Staying Well Map’ as this collaborative process made the therapy feel personalised, with students feeling a sense of ownership over their map. Although the collaborative element of creating the map was not lost in context of remote working, students and practitioners acknowledged that this process would have felt more collaborative in-person. It is therefore important for this aspect of the therapy to feel as collaborative as possible. As the therapists did not feel comfortable using the online software to construct the map, further training in digital know-how is warranted if the service is to continue remotely. Further training around identifying non-verbal cues in the online milieu would also be fruitful. 

## 5. Conclusions

To conclude, the provision of brief psychotherapy to students with self-harm-related difficulties is promising. Students appreciated the rapid access to intervention, especially as student services are typically characterized by long waiting lists. Rapid access to intervention may help prevent deterioration and worsening difficulties. Despite the brief nature of the intervention, therapeutic alliances sufficient to facilitate psychological change appeared possible online, and many students reported feeling a sense of control over the direction and pace of the therapeutic sessions. Although some students appreciated the opportunity to engage remotely, others would have preferred face-to-face provision. Thus, a ‘one size fits all’ strategy is not appropriate in the aftermath of the pandemic. In considering future models of provision, assessing clinical need, student preference, and access to space are important considerations when deciding which modality to achieve the best outcomes for each individual and by session. While the results reported here suggest that U-COPE is helpful to this population, a larger-scale evaluation of this brief intervention is called for due to the small sample size.

## Figures and Tables

**Table 1:** Overarching themes and subthemes.

Themes	Subthemes
Accessibility	‘Quick’ and ‘easy’ referrals and access
	Remote access ‘made it more accessible’
Therapeutic experiences	Therapeutic alliance
	Collaboration: ‘It’s not quite the same as working in-person’
	Efficacy
Spaces and places of therapy	Spaces of comfort and safety
	Moving the emotions on

## Data Availability

Qualitative data extracts are presented in the article to support the findings. The data generated and analysed during the current study are not publicly available as the data collected are sensitive and could compromise the confidentiality and anonymity of the participants but are available (limited) from the corresponding author on reasonable request.
